# The Appropriate Marker for Astrocytes: Comparing the Distribution and Expression of Three Astrocytic Markers in Different Mouse Cerebral Regions

**DOI:** 10.1155/2019/9605265

**Published:** 2019-06-24

**Authors:** Zengli Zhang, Zhi Ma, Wangyuan Zou, Hang Guo, Min Liu, Yulong Ma, Lixia Zhang

**Affiliations:** ^1^Department of Anesthesiology, Xiangya Hospital, Central South University, Changsha 410008, China; ^2^Department of Anesthesiology, Center for Brain Science, The First Affiliated Hospital of Xi'an Jiaotong University, Xi'an 710061, China; ^3^Department of Anesthesiology, The Seventh Medical Center to Chinese PLA General Hospital, Beijing 100700, China; ^4^Anesthesia and Operation Center, The First Medical Center to Chinese PLA General Hospital, Beijing 100853, China; ^5^Department of Burn and Plastic Surgery, The Fourth Medical Center to Chinese PLA General Hospital, Beijing 100048, China

## Abstract

Astrocytes possess different morphological characteristics depending on the cerebral region in which they are found. However, none of the current astrocytic markers can label all subpopulations successfully. Thus, identifying the appropriate marker for a specific scientific investigation is critical. Here, we compared the distribution and protein expression of three astrocyte markers: NDRG2, GFAP, and S100*β*, in the cortex, hippocampus, and thalamus. NDRG2- and S100*β*-positive astrocytes were distributed more uniformly than GFAP-positive astrocytes throughout the whole cerebrum. NDRG2 and S100*β* immunoreactivities were the strongest in the dorsal cortex and thalamus, while GFAP immunoreactivity was the strongest in the hippocampus. Moreover, protein expression levels of NDRG2, GFAP, and S100*β* in adult mice were the highest in the cortex, hippocampus, and thalamus, respectively. We also detected astrocyte morphology and found that, in the corpus callosum and cerebral peduncle, GFAP-positive astrocytes were found with more numerous and longer processes than NDRG2- and S100*β*-positive astrocytes. These results demonstrate that NDRG2 and S100*β* are more suitably used to visualize the overall distribution and changes in the number of astrocytes, as well as label astrocytes in the cortex and thalamus. GFAP, however, is more appropriately used to label astrocytes in the corpus callosum, cerebral peduncle, and the hippocampus. These results help to guide researchers in the choice of appropriate astrocyte marker and suggest differences in immunological qualities of astrocytes based on the tissue in which they are found.

## 1. Introduction

Astrocytes have long been considered auxiliary cells that provide only trophic, metabolic, and structural support to neurons [1]. However, in recent years, extensive research has shown that they play crucial roles in cerebral physiological and pathological functions. Astrocytes help in the maintenance of ion homeostasis and blood-cerebral barriers, synapse formation, and removal, as well as controlling cerebral blood flow and regulating neurotransmitter recycling, glutamate excitotoxicity, and antioxidant stress [2–4]. Importantly, astrocytes possess morphological, population, and functional diversities in different cerebral regions [2, 5]. Therefore, identifying the most appropriate marker for the polymorphous and multiple subgroups of astrocytes is critical to research on astrocytic multifunction.

Glial fibrillary acidic protein (GFAP) is the most commonly used astrocytic marker, but as the major intermediate filament composing cytoskeleton, GFAP immunolabeled only about 15% of the total astrocyte volume [6], and more than 40% of astrocytes were found to be GFAP-negative in the adult rat hippocampus [7]. Additionally, GFAP labelled protoplasmic human astrocytes poorly and was expressed late in development of fibrous astrocytes [1, 8].

Another commonly used astrocytic marker is S100*β*, a Ca^2+^ binding peptide abundant in the cytoplasm, and nucleus of astrocytes that is involved in cell cycle regulation and cytoskeleton modification [9]. However, S100*β* is also expressed in a subpopulation of mature oligodendrocytes, in the choroid plexus epithelial cells, and in a few neurons [10, 11]. The deficiencies of GFAP and S100*β* in labeling astrocytes may lead to inaccuracies or even mistakes in exploring the functions of astrocytes.

N-Myc downstream-regulated gene 2 (NDRG2) was first discovered in a normal human cerebral cDNA library by a PCR-based subtractive hybridization method [12]. It is a tumor suppressor and cell stress-related gene associated with cell proliferation and differentiation [13]. NDRG2 is widely expressed in the cerebral cortex, olfactory bulb, midcerebral, hippocampus, and thalamus [14]. Most importantly, NDRG2 is specifically expressed in astrocytes of the brain [14–16]. Thus, NDRG2 is regarded as a novel astrocytic marker, especially for mature, nonreactive, and nonproliferating astrocytes [15]. However, whether NDRG2 is more reliable than GFAP and S100*β* as an astrocytic marker, as well as the differences of their distribution and expression in different cerebral regions, has not been reported.

In the current study, we compared the distribution, protein expression, and mutual colocalization of astrocytic markers NDRG2, GFAP, and S100*β* throughout the whole cerebrum of mice. We aimed to identify the most suitable marker for astrocytes in different cerebral regions.

## 2. Materials and Methods

### 2.1. Animals

Young, adult, and old* C57BL/6* male mice aged 1 month, 3 months, 6 months, 9 months, and 12 months were obtained from the Experimental Animal Center of the Central South University. The animals were free to eat and drink, kept at a temperature of 25 ± 1°C, and housed with a 12:12-h light-dark circle to simulate the animal's circadian rhythm. All efforts were made to minimize animal suffering and to reduce the number of animals used. All animal experimental procedures followed a protocol approved by the Ethical Committee for Animal Experimentation of the Central South University Animal Care and Use Committee, China.

### 2.2. Cell Cultures

The HT22 cells (a neuronal cell line) and MA1800-57 cells (an astrocyte cell line) were cultured in Dulbecco's Modified Eagle's Medium (DMEM, HyClone) containing 10% fetal bovine serum (FBS, Gibco), 50 U/mL penicillin, and 50 *μ*g/mL streptomycin. The OLN-93 cells (an oligodendroglial cell line) were kept in DMEM supplemented with 10% FBS, 2 mM Glutamine, 50 U/mL penicillin, and 50 *μ*g/mL streptomycin. The cells were all maintained at 37°C in a mixture of 95% atmospheric air and 5% CO2. The HT22 cells, OLN-93 cells, and MA1800-57 cells were seeded on slides and were stained with antibodies against microtubule-associated protein 2 (MAP2), *α*-tubulin, and GFAP, respectively.

### 2.3. Immunofluorescence Staining Assay

After the mice were anesthetized with sodium pentobarbital (50mg/kg), their cerebrums were extracted and fixed with 0.9% cold heparinized saline and 4% paraformaldehyde. After postfixation, the cerebrums were successively dehydrated in 20% sucrose and 30% sucrose solutions. Sections of 10 *μ*m thickness were prepared using a Leica CM1900 frozen slicer. The cerebral sections were incubated in 1% H_2_O_2_ for 15 min and 0.3% Triton X-100 for 15 min, with 3×washes in PBS postincubation between each treatment. The sections were then blocked in 5% normal goat serum for 1 h at room temperature and incubated overnight at 4°C in a humidifying box with primary antibodies as follows: mouse anti-GFAP antibody (#3670, 1:500, Cell Signaling Technology); rabbit anti-NDRG2 antibody (#5667, 1:200, Cell Signaling Technology); mouse anti-NDRG2 antibody (#DA281-6A5, 1:100, Sigma); rabbit anti-S100*β* antibody (#2017-1, 1:500, Epitomics); mouse anti-Glutamine Synthetase (GS) antibody (#MAB302, 1:200, Millipore); rabbit anti-MAP2 antibody (#ab32454, 1:500, Abcam); and mouse anti-*α*-tubulin antibody (#T6199, 1:500, Sigma). Then, the sections were incubated with mixtures of Alexa-488 (green, Invitrogen) and Alexa-647 (red, Invitrogen)-conjugated donkey anti-rabbit or donkey anti-mouse secondary antibodies for 2 h in the dark at room temperature. 4,6-Diamidino-2-phenylindole (DAPI) (ZLI-9557, Zsbio) was used to stain nuclei. The sections were mounted with 50% glycerol. Finally, the sections were viewed and photographed using an Olympus BX51 (Japan) fluorescence microscope. The antibodies used in our study were widely used in our previous studies and by other investigators [17–20], and the sensitivity and specificity of these antibodies have been confirmed.

### 2.4. Western Blot

The cortex, hippocampus, and thalamus were collected from C57BL/6 mice aged 1 month, 3 months, 6 months, 9 months, and 12 months. The samples were homogenized in RIPA buffer, and the concentration of the protein samples was measured by BCA Protein Assay Kit (Pierce Biotechnology, US). The protein samples were separated by 10% SDS-PAGE (SDS-PAGE Gel kit, CW0022S, China) and electrically transferred to polyvinylidene difluoride (PVDF) membranes. The components of the SDS-PAGE Gel kit included a 30% Acr-Bis, SDS-PAGE Separating Gel Buffer, SDS-PAGE Stacking Gel Buffer, APS (ammonium persulfate), and TEMED (N, N, N′, N′-tetramethylethylenediamine). Subsequently, the membranes were blocked in 5% nonfat dry milk diluted in TBST (TBS with 0.05% Tween 20) for 1 h at room temperature and then incubated with specific primary antibodies: mouse anti-GFAP antibody (#3670, 1:1000, Cell Signaling Technology); rabbit anti-NDRG2 antibody (#5667, 1:1000, Cell Signaling Technology); rabbit anti-S100*β* antibody (#2017-1, 1:1000, Epitomics); and rabbit anti-GAPDH antibody (#5174, 1:1000, Cell Signaling Technology) overnight at 4°C. The membranes were then incubated with an HRP-conjugated secondary anti-rabbit or anti-mouse antibody (Thermo Scientific, USA) for 2 h. Chemiluminescent signals were developed using Western Lightning Chemiluminescence Reagent Plus (PerkinElmer Life Sciences; Wellesley, MA) and detected by an image analyzer LI-COR Odyssey System (LI-COR Biotechnology, USA). The immunoreactivity of proteins bands was quantitatively analyzed by Bio-Rad® Image Lab™ software. Band density values were normalized to GAPDH.

### 2.5. Quantitative (Real-Time) Polymerase Chain Reaction (Real-Time PCR)

The cortex, hippocampus, and thalamus were collected from* C57BL/6* mice aged 1 month, 3 months, 6 months, 9 months, and 12 months. Total RNA was isolated using a TransZol Up Plus RNA Kit (Code No. ER501-01, Transgen, China) and quantified using a NanoDrop 2000. Then, 1000 ng of total RNA was reverse transcribed in a 20 *μ*L reaction at 42°C for 15 min, followed by 85°C for 5 sec using a* TransScript*® All-in-One First-Strand cDNA Synthesis SuperMix for qPCR (Code No. AT341, Transgen, China). The components of the kit included a 5×*TransScript*® All-in-One SuperMix for qPCR (TransScript® RT, RNase Inhibitor, Anchored Oligo(dT)18 Primer, Random Primer(N9), dNTPs, buffer), a 5×*TransScript*® All-in-One No-RT Control SuperMix for qPCR, a gDNA Remover and a RNase-free water. The 5×*TransScript*® All-in-One No-RT Control SuperMix for qPCR was used as a negative control. Real-time PCR was performed in a 20 *μ*L reaction according to the manufacturer's manual for TransStart Tip Green qRNA SuperMix (Code No. AQ141, Transgen, China). The mRNA primer sequences used can be found in Table 1. The reaction was performed at 94°C for 30 sec followed by 40 cycles of 94°C for 5 sec, 60°C for 15 sec, and 72°C for 19 sec on the ViiA7 Real-Time PCR Detection System (2720 Thermal Cycler, Applied Biosystems). The primers and sequences derived from the relative mRNA expression were analyzed using the formula 2^-(Ct  target  gene-Ct  reference  gene)^.

### 2.6. Statistical Analysis

Statistical tests were performed using PRISM v7.0 software (GraphPad). Comparisons between two groups were performed using Student's* t*-tests. Comparisons between various groups were performed using one-way ANOVA with Tukey's posttest. All values were presented as the mean ± SD. Values of* p*<0.05 were considered statistically significant.

## 3. Results

### 3.1. The Distribution of Astrocytes Labeled by NDRG2, GFAP, and S100*β* in Different Cerebral Regions

**Figure 1:**
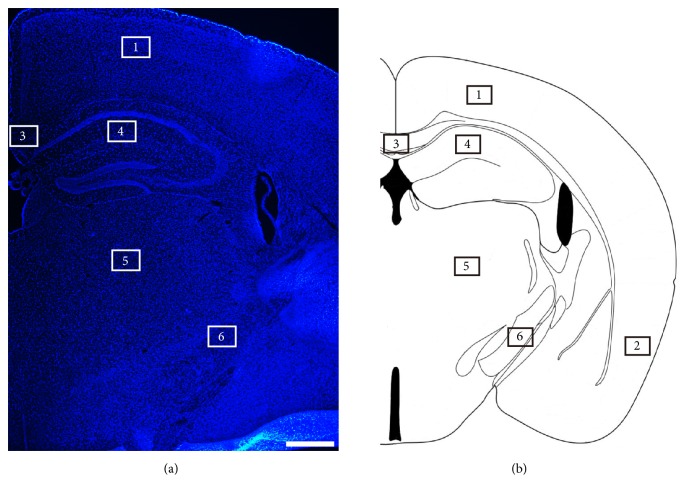
*The anatomical regions of the mice cerebrum*. (a) Representative immunofluorescence images of the cells labeled by DAPI in the cerebrum of the male mice. Scale bars = 500 *μ*m. (b) The anatomic atlas of the mouse cerebrum. Region 1: dorsal cortex; Region 2: ventral cortex; Region 3: corpus callosum; Region 4: hippocampus; Region 5: thalamus; Region 6: cerebral peduncle.

**Figure 2:**
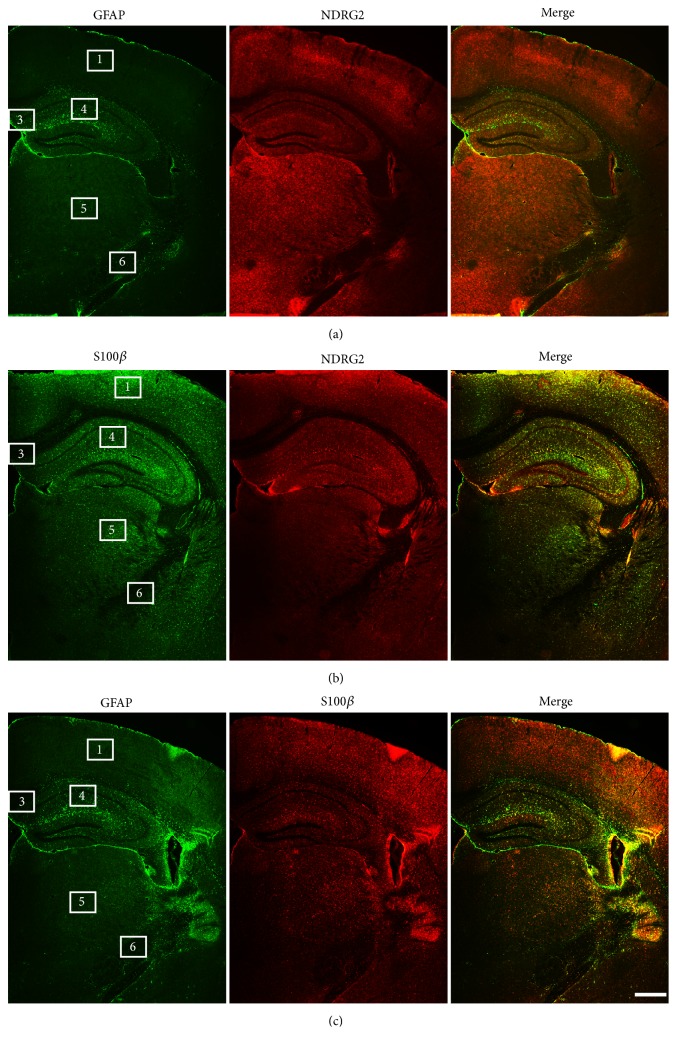
*The distribution of astrocytes labeled by NDRG2, GFAP and S100β throughout the cerebrum*. Representative immunofluorescence images of astrocytes labeled by GFAP and NDRG2 (a), S100*β*, and NDRG2 (b), GFAP and S100*β* (c) in the cerebrum of the adult male mice (6 months) at low magnification. Region 1: dorsal cortex; Region 2: ventral cortex; Region 3: corpus callosum; Region 4: hippocampus; Region 5: thalamus; Region 6: cerebral peduncle. n = 6. Scale bars = 500 *μ*m.

The regions of the mice cerebra were identified by labeling the cell nuclei. Additionally, a corresponding mouse brain atlas was used to verify the specific regions analyzed (Figure 1). We then used immunofluorescence staining to detect the distribution of astrocytes labeled by NDRG2, GFAP, and S100*β* in different cerebral regions of adult male mice aged 6 months. NDRG2- and S100*β*-positive astrocytes were more abundant and more evenly distributed than GFAP-positive astrocytes throughout the whole cerebrum (Figure 2). NDRG2-positive astrocytes were concentrated in the dorsal cortex (Region 1) and thalamus (Region 5), but less so in the hippocampus (Region 4), corpus callosum (Region 3), and ventral cortex (Region 2) and the least in the cerebral peduncle (Region 6) (Figures 2(a) and 2(b)). GFAP-positive astrocytes were concentrated most in hippocampus; less were detected in the corpus callosum and cerebral peduncle and almost undetectable in the dorsal cortex, ventral cortex, and thalamus (Figures 2(a) and 2(c)). S100*β*-positive astrocytes were concentrated most in the dorsal cortex and thalamus, less so in the hippocampus and ventral cortex, and least of all in the cerebral peduncle and corpus callosum (Figures 2(b) and 2(c)). The distribution of the NDRG2-positive astrocytes was similar to that of the S100*β*-positive astrocytes.

Astrocytes in different cerebral regions presented unequable immunoreactivities to NDRG2, GFAP and S100*β* (Figure 2). Astrocytes in the dorsal cortex and thalamus reacted strongly to NDRG2, and S100*β*, but weakly to GFAP. Astrocytes in the hippocampus reacted strongly to GFAP and moderately to NDRG2 and S100*β*. Astrocytes in the ventral cortex, corpus callosum, and cerebral peduncle reacted weakly to moderately to NDRG2, GFAP, and S100*β* (Table 2).

**Figure 3:**
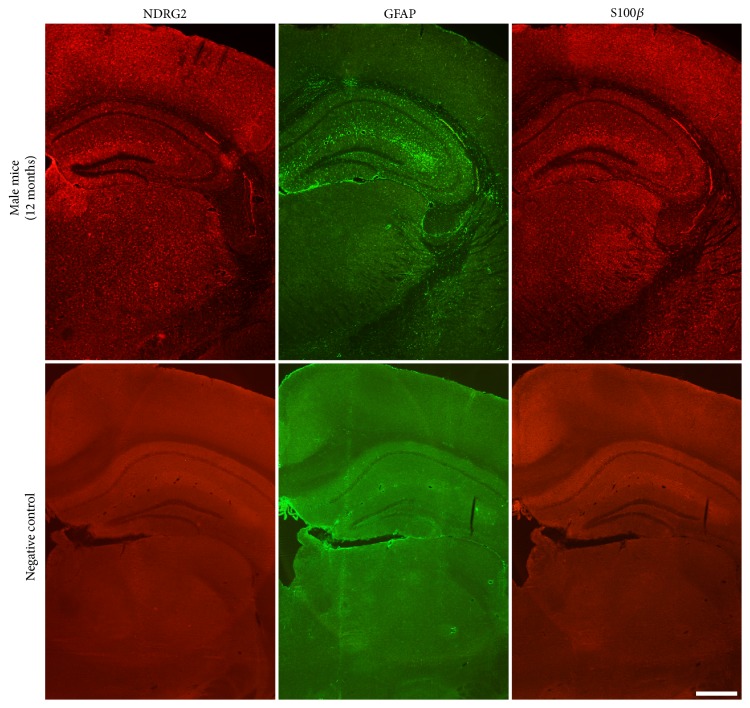
*The distribution of astrocytes labeled by NDRG2, GFAP and S100β in the cerebrum*. Representative immunofluorescence images of astrocytes labeled by NDRG2, GFAP, and S100*β* in the cerebrum of the old male mice (12 months) and the negative control. Scale bars = 500 *μ*m.

The distribution of astrocytes labeled by NDRG2, GFAP, and S100*β* in different cerebral regions of male mice was similar to that of old male mice (12 months) (Figure 3).

### 3.2. The Morphologies of Astrocytes Labeled by NDRG2, GFAP, and S100*β* in Different Cerebral Regions

**Figure 4:**
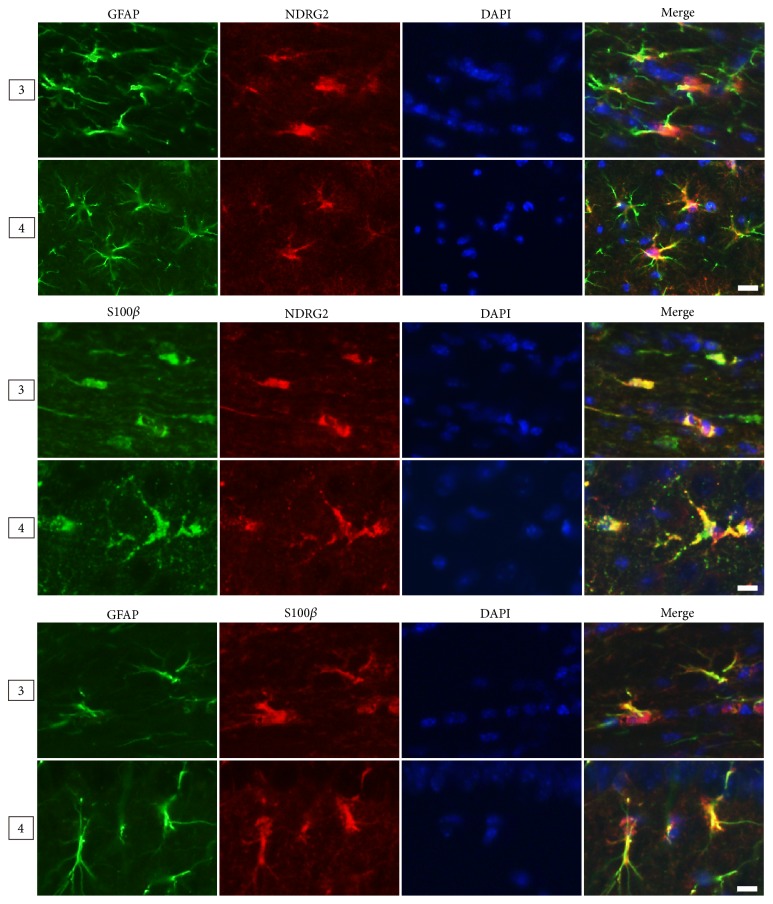
*The morphology of astrocytes labeled by NDRG2, GFAP and S100β in different cerebral regions*. Representative immunofluorescence images of astrocytes labeled by NDRG2, GFAP, and S100*β* in the cerebrum of the adult male mice (6 months) at high magnification. Region: corpus callosum; Region 4: hippocampus. Scale bars = 10 *μ*m.

**Figure 5:**
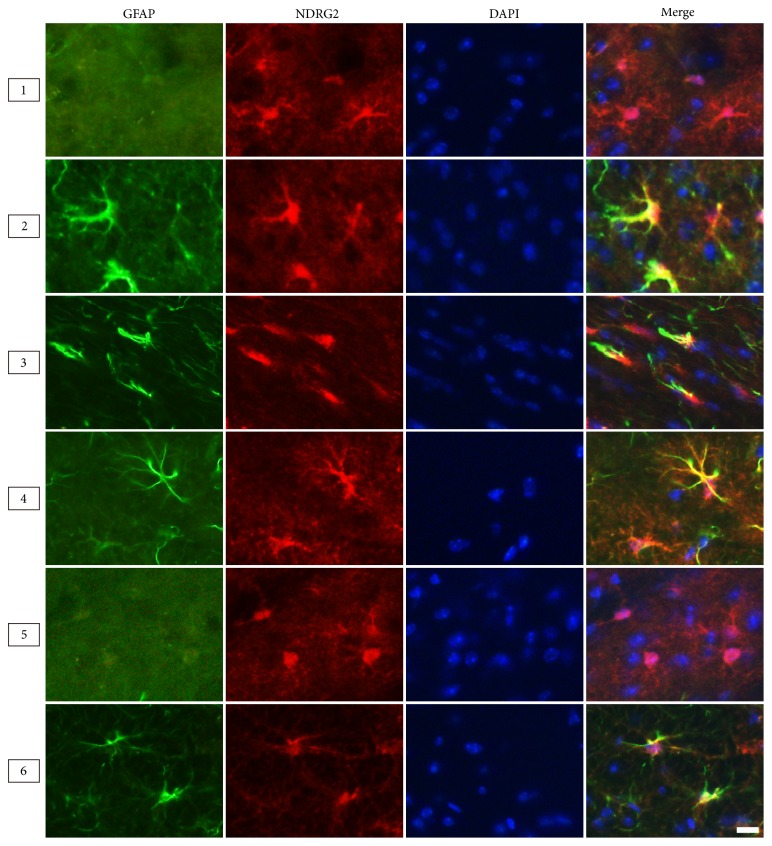
*The colocalization of NDRG2 and GFAP within astrocytes in different cerebral regions*. Representative immunofluorescence images of astrocytes labeled by NDRG2 and GFAP in the cerebrum of the adult male mice (6 months). Region 1: dorsal cortex; Region 2: ventral cortex; Region 3: corpus callosum; Region 4: hippocampus; Region 5: thalamus; Region 6: cerebral peduncle. Scale bars = 10 *μ*m.

Astrocytes are mainly divided into two types: fibrous astrocytes in white matter and the protoplasmic astrocytes in gray matter. Therefore, we compared the morphologies of the two types labeled by different markers in the corpus callosum (white matter, Region 3) and the hippocampus (gray matter, Region 4) (Figure 4). The results from adult male mice (6 months) showed the NDRG2- and S100*β*-positive astrocytes presented a stellate shape characterized by large and round soma along with short and coarse cytoplasmic processes that had few branches. The GFAP-positive astrocytes presented with a radial shape characterized by small soma, long processes, and multibranches (Figure 4). Moreover, in the corpus callosum and cerebral peduncle, the astrocytes labelled by GFAP presented with more abundant longer processes than those labelled by NDRG2 and S100*β* (Figures 5 and 7).

### 3.3. The Colocalization of NDRG2, GFAP, and S100*β* within Astrocytes in Different Cerebral Regions

**Figure 6:**
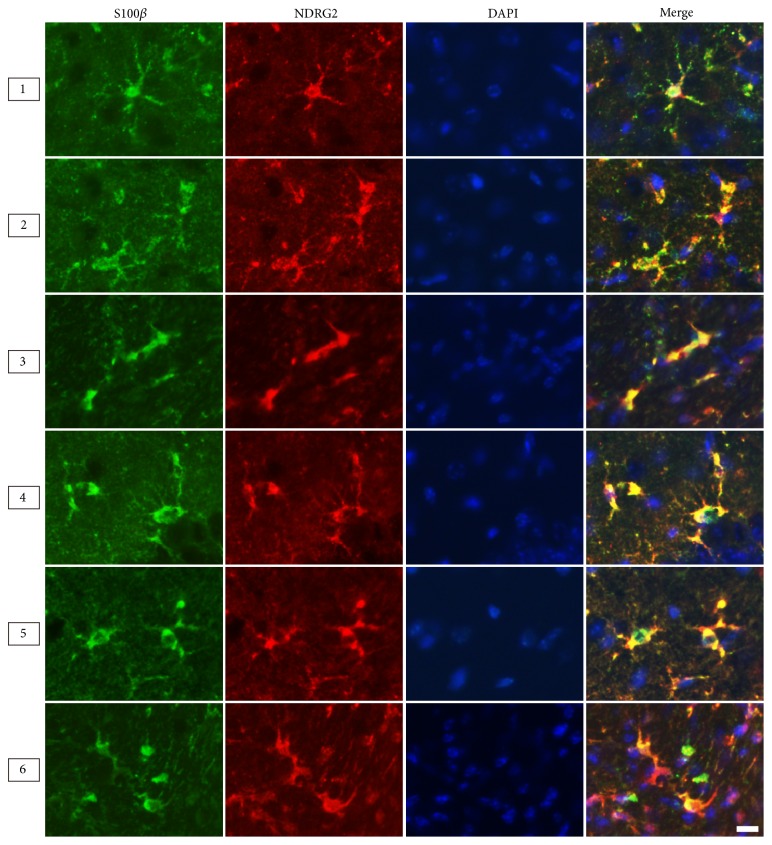
*The colocalization of NDRG2 and S100β within astrocytes in different cerebral regions*. Representative immunofluorescence images of astrocytes labeled by NDRG2 and S100*β* in the cerebrum of the adult male mice (6 months). Region 1: dorsal cortex; Region 2: ventral cortex; Region 3: corpus callosum; Region 4: hippocampus; Region 5: thalamus; Region 6: cerebral peduncle. Scale bars = 10 *μ*m.

**Figure 7:**
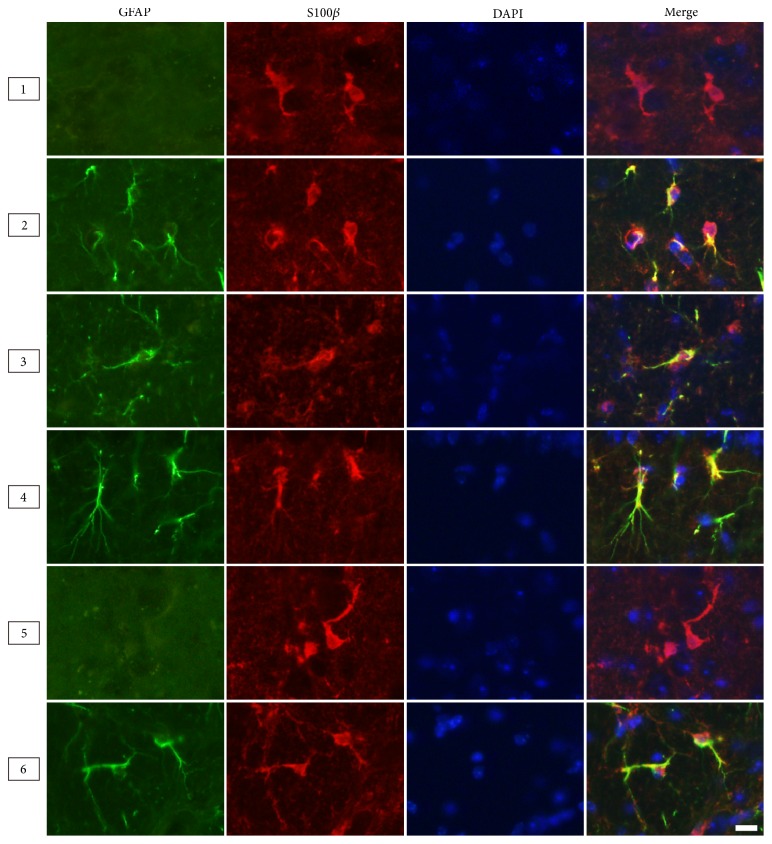
*The colocalization of GFAP and S100β within astrocytes in different cerebral regions*. Representative immunofluorescence images of astrocytes labeled by GFAP and S100*β* in the cerebrum of the adult male mice (6 months). Region 1: dorsal cortex; Region 2: ventral cortex; Region 3: corpus callosum; Region 4: hippocampus; Region 5: thalamus; Region 6: cerebral peduncle. Scale bars = 10 *μ*m.

**Figure 8:**
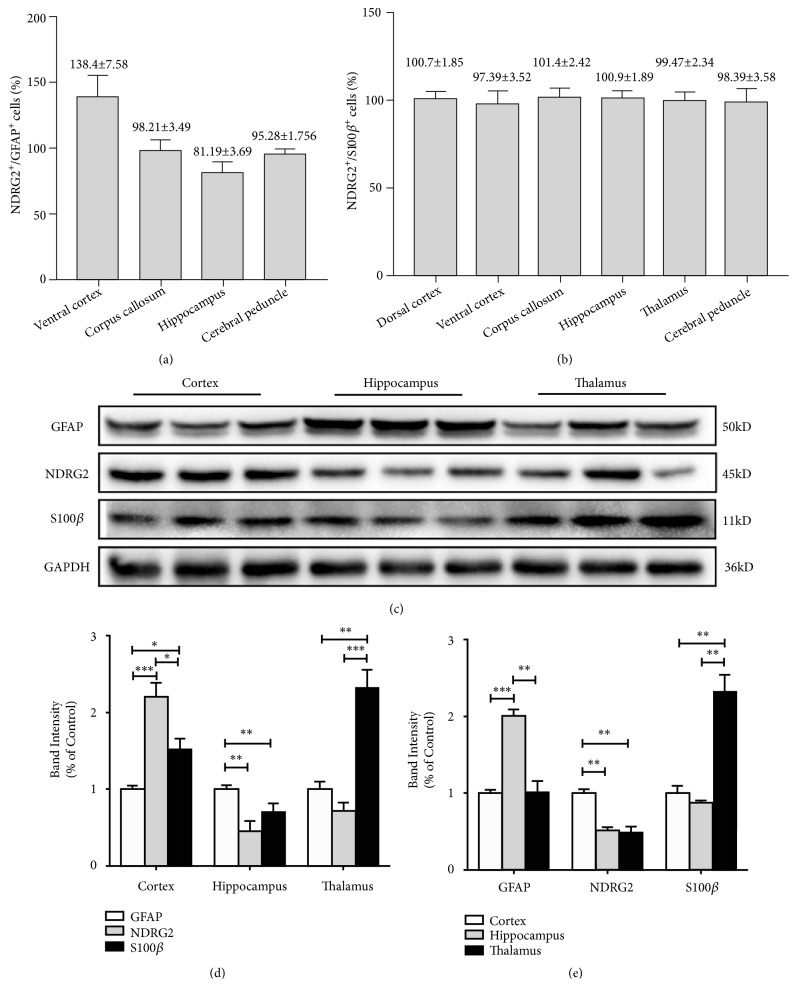
*The colocalization and protein expression of NDRG2, GFAP, and S100β in different cerebral regions*. (a)-(b) The quantitative analysis of NDRG2^+^/GFAP^+^ astrocytes and NDRG2^+^/S100*β*^+^ astrocytes. Results were presented as the mean ± SD. Cells in five fields were counted. (c)-(e) The expression levels of NDRG2, GFAP, and S100*β* proteins in cortex, hippocampus and thalamus of the adult male mice (6 months) were determined by Western blot analysis. Representative blots and quantitative analysis were shown. Results were presented as the mean ± SD and analyzed by one-way ANOVA with Tukey's posttests. n = 6. ^*∗*^*p*<0.05, ^*∗∗*^*p*<0.01, and ^*∗∗∗*^*p*<0.001.

NDRG2 was nearly colocalized with GFAP in astrocytes of the ventral cortex, corpus callosum, and cerebral peduncle, and mostly colocalized with GFAP in astrocytes of the hippocampus (Figures 5 and 8(a)). NDRG2 had nearly no colocalization with GFAP in astrocytes of the dorsal cortex and thalamus (Figure 5). NDRG2 and S100*β* presented nearly complete colocalization in astrocytes of the six cerebral regions (Figures 6 and 8(b)). GFAP and S100*β* presented with significant colocalization in astrocytes of the ventral cortex, corpus callosum, and cerebral peduncle and nearly complete colocalization in astrocytes of the hippocampus (Figure 7). GFAP and S100*β* had nearly no colocalization in astrocytes of the dorsal cortex and thalamus (Figure 7).

We also detected colocalization of NDRG2 and another astrocytic maker, glutamine synthetase (GS), in astrocytes of the mice cerebrum. NDRG2 and GS had significant colocalization in astrocytes (Figure s1a). NDRG2 and GS presented thorough colocalization in fibrous astrocytes of the corpus callosum and in protoplasmic astrocytes of the hippocampus (Figure s1b). NDRG2 protein expression in the neuronal cell line HT22 cells, oligodendroglial cell line OLN-93 cells, and astrocytic cell line MA1800-57 cells were also detected (Figure s2). NDRG2 protein was detected in MA1800-57 cells and was barely detected in HT22 cells and OLN-93 cells (Figures s2a and s2b).

### 3.4. The Expression of NDRG2, GFAP, and S100*β* Proteins in Different Cerebral Regions

We used western blotting to detect the expression levels of NDRG2, GFAP and S100*β* proteins in three cerebral regions of adult male mice (6 months): the cortex, hippocampus, and thalamus (Figure 8(c)). We analyzed the expression levels of these markers in the same cerebral region (Figure 8(d)). In the cortex, the level of NDRG2 expression was markedly higher than GFAP and S100*β* expression levels (^*∗∗∗*^*p*<0.001, ^*∗*^*p*<0.05). The expression level of S100*β* was also much higher than GFAP (^*∗*^*p*<0.05). In the hippocampus, the level of GFAP expression was higher than NDRG2 and S100*β* (^*∗∗*^*p*<0.01), and the level of NDRG2 expression was a little less than the S100*β* expression level, although the difference was not statistically significant. In the thalamus, the expression level of S100*β* was significantly higher than GFAP and NDRG2 expression levels (^*∗∗*^*p*<0.01), while the level of NDRG2 expression was similar to that of GFAP.

Next, we analyzed the expression levels of the same marker in different cerebral regions (Figure 8(e)). The level of NDRG2 expression in the cortex was much higher than in the hippocampus and thalamus (^*∗∗*^*p*<0.01), and no significant difference was observed between the hippocampus and thalamus. The level of GFAP expression in the hippocampus was the highest among the three regions (^*∗∗∗*^*p*<0.001, ^*∗∗*^*p*<0.01); no significant difference was observed between the cortex and thalamus. The level of S100*β* expression in the thalamus was significantly higher than in the cortex and hippocampus (^*∗∗*^*p*<0.01), with no significant difference observed between the latter two.

**Figure 9:**
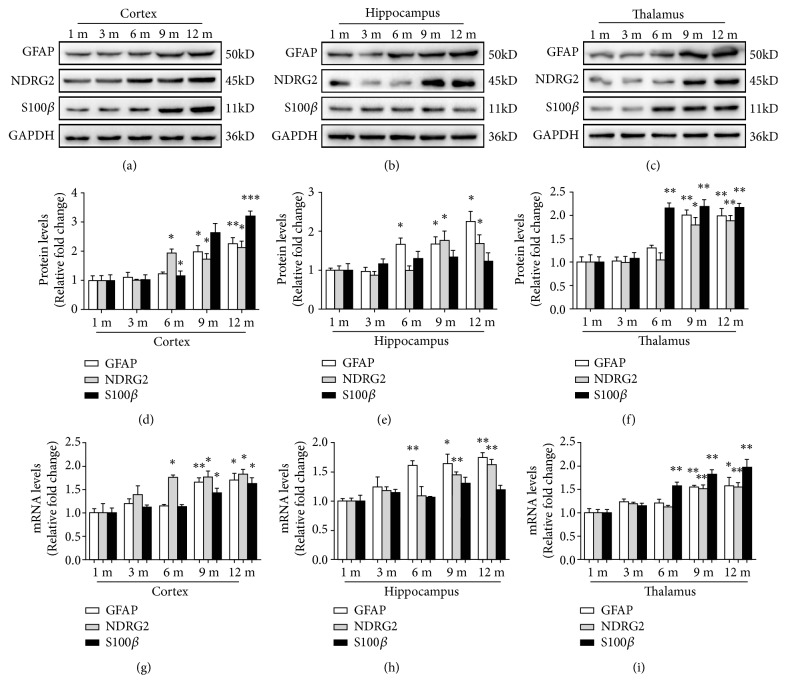
*The expression of NDRG2, GFAP, and S100β in different cerebral regions of different aged male mice*. (a)-(f). The levels of NDRG2, GFAP, and S100*β* protein in cortex, hippocampus, and thalamus of the male mice aged 1 month, 3 months, 6 months, 9 months, and 12 months were determined by Western blot analysis. Representative blots and quantitative analysis were shown. (g)-(i) The levels of NDRG2, GFAP and S100*β* mRNA in cortex, hippocampus, and thalamus of the male mice aged 1 month, 3 months, 6 months, 9 months, and 12 months were determined by qPCR analysis. The quantitative analysis was shown. The mean of 1 month was standardized to 1. Results were presented as the mean ± SD and analyzed by Student's* t*-tests. n=6. ^*∗*^*p*<0.05, ^*∗∗*^*p*<0.01, and ^*∗∗∗*^*p*<0.001. 1 m = 1 month; 3 m = 3 months; 6 m = 6 months; 9 m = 9 months; 12 m = 12 months.

We also detected the protein levels of NDRG2, GFAP, and S100*β* in the cortex, hippocampus, and thalamus of male mice aged 1 month, 3 months, 6 months, 9 months, and 12 months (Figures 9(a)–9(f)). The protein levels of NDRG2 and GFAP in the cortex, hippocampus, and thalamus gradually increased with age (^*∗*^*p*<0.05, ^*∗∗*^*p*<0.01). Protein levels of S100*β* in the cortex and thalamus, but not in the hippocampus, gradually increased with age (^*∗*^*p*<0.05, ^*∗∗*^*p*<0.01, ^*∗∗∗*^*p*<0.001). The mRNA levels of NDRG2, GFAP, and S100*β* in the cortex, hippocampus and thalamus of male mice aged 1 month, 3 months, 6 months, 9 months, and 12 months were consistent with those of the protein levels (^*∗*^*p*<0.05, ^*∗∗*^*p*<0.01, Figures 9(g)–9(i)).

## 4. Discussion

In this study, we found that astrocytes labeled by NDRG2 and S100*β* were distributed more widely and uniformly than those labeled by GFAP in the whole cerebrum of young, mature, and old mice. This suggests that NDGR2 and S100*β* are more appropriate markers to observe the overall distribution and the number changes of astrocytes. Moreover, our results showed that astrocytes in different cerebral regions presented different levels of immunoreactivity to NDRG2, GFAP, and S100*β*, which suggests that, in the cortex and thalamus, NDRG2 and S100*β* are more appropriate astrocytic markers than GFAP. The unequable immunoreactivity of astrocytes to NDRG2, GFAP, and S100*β* might also be due to the sensitivity and specificity of the antibodies we used. Additionally, the morphologies of astrocytes labeled by NDRG2 and S100*β* were similar to each other, but were obviously distinct from those labeled by GFAP because these markers labeled different cytoskeletal structures. NDRG2 stains the cytoplasm and cell membranes and weakly stains the nucleus [21]. GFAP mainly stains the soma and processes, but not the nucleus, while S100*β* stains the nucleus and part of the cytoplasm and processes [22]. By comparing the morphologies of astrocytes labeled by NDRG2, GFAP, and S100*β*, we found that GFAP was a more suitable marker for astrocytes in the corpus callosum, cerebral peduncle, and especially the hippocampus. The proteins NDRG2 and S100*β* were expressed most in the cortex and thalamus, respectively. We infer that NDRG2 is more suitable for astrocytes in the cortex while S100*β* is more suitable for astrocytes in thalamus when quantifying astrocytic density. The different protein expression patterns of NDRG2, GFAP, and S100*β* in the cortex, hippocampus, and thalamus confirmed astrocytic functional heterogeneity.

Astrocytes have been found to play an important role in the maintenance of homeostasis, and they actively participate in almost all neurological disorders, such as ischemic strokes, traumatic cerebral injuries, Alzheimer disease, and Parkinson's disease [10, 11, 23]. It has been demonstrated that astrocytic morphology, gene expression, and function differed among different regions of the cerebrum [24, 25]. Fibrous astrocytes and protoplasmic astrocytes are two major subpopulations identified by their morphology [24, 25]. Fibrous astrocytes have long, thin processes, and a star-like appearance, while the protoplasmic ones have numerous fine processes, which contact and sheath synapses [25]. Interestingly, many genes are heterogeneously expressed by subsets of astrocytes. These genes encode proteins such as glutamate transporters [26, 27] and ion channels [28], as well as glutamate [29], dopamine [30], and opioid receptors [31].

The heterogeneity of gene expression implied the functional diversity of astrocytes. For example, glutamate uptake differs among different subsets [32]. Additionally, spontaneous calcium oscillations differ among different layers of the somatosensory cortex. Cortical layer 1 astrocytes showed frequent asynchronous Ca^2+^ activity, whereas layer 2/3 astrocytes showed infrequent synchronous Ca^2+^ activity [33]. In the cortex, astrocytes respond to glutamate and norepinephrine with increases in calcium, while hippocampal astrocytes exhibit calcium responses to ATP, GABA, glutamate, acetylcholine, prostaglandins, and endocannabinoids [34]. Studies have also shown that cultured astrocytes from different brain regions have different capacities to stimulate neurite growth and branching [35]. As astrocytes in different brain regions present different characteristics of morphology and function, identifying the most appropriate marker for astrocytes in different cerebral regions is crucial for astrocytic researchers.

Although several proteins have been reported as selectively expressed by astrocytes, none have been shown to be entirely restricted to all astrocytic subtypes. GFAP, the most widely used astrocytic marker, is preferentially expressed in white matter astrocytes over gray matter ones and fails to label all the processes of astrocytes [6, 23]. More importantly, there are subsets of GFAP-negative astrocytes that present voltage-dependent K^+^ currents, whereas the GFAP-positive astrocytes present the classical pattern of time- and voltage-independent currents [7, 36, 37].

Previous studies found that S100*β* was able to label astrocytes in both gray and white matter; however, it was also expressed in a few oligodendrocytes and neurons [11]. S100*β* was found to be expressed most in thalamus astrocytes, a subtype of cells that participate in the cleaning of DAergic debris produced by the degeneration of DAergic terminals in Parkinson's disease by facilitating the presence of lysosomes and the formation of autophagosomes [38]. The transcriptomic analysis of astrocytes identified aldehyde dehydrogenase 1 family, member L1 (ALDH1L1), as an astrocytic marker [23]. However, ALDH1L1 also labeled oligodendrocytes [39]. Similarly, the excitatory amino acid transporters glutamate/aspartate transporter (GLAST), glutamine synthetase (GS), glutamate transporter-1(GLT-1), vimentin, connexin 43, aquaporin 4, and the cell surface glycoprotein CD44 also had limitations when labeling astrocytes [7, 40–44].

NDRG2 is specifically expressed in astrocytes of both rats and mice [15, 16] and is associated with the growth and maturation of the developing embryonic brain [45]. In addition to cell proliferation, differentiation, and transmembrane transports, NDRG2 also participates in stress responses, depression, ischemic diseases, and neurodegenerative disorders [43, 45]. In mice and humans, NDRG2 participates in the development of attention-deficit/hyperactivity disorder (ADHD), a disease associated with the locomotion center in the cerebral cortex [18]. Flugge et al. detected the expression of NDRG2 in the cerebrums of humans, marmosets, tree shrews, rats, and mice, and suggested NDRG2 was a new astrocytic marker, especially for mature, nonreactive, and nonproliferating astrocytes [16]. In this study, we first detected the distribution pattern of NDRG2-positive astrocytes in different cerebral regions and differences with the astrocytes labeled by classic markers GFAP and S100*β*.

Astrocytes and astrocytic markers changed during normal brain aging. Previous work has shown that astrocytes are activated early in aging, and the significant increase of GFAP-positive astrocytes was found in the hippocampal regions of aged mice [1, 46]. Levels of both GFAP protein and GFAP mRNA increased in various parts of aging brains, such as the cerebral cortex, hippocampus, and cerebellum [47]. Discrepancies remain among various reports of S100*β* in the aging brain [48]. Increasingly, studies have shown that NDRG2 is associated with age-related disorders such as Alzheimer's disease. In our study, levels of GFAP and NDRG2 mRNA in the cortex, hippocampus, and thalamus gradually increased with age, while levels of S100*β* mRNA in the hippocampus and thalamus increased with age, but not in the cortex.

We should note that, currently, many markers in addition to the ones we tested, such as ALDH1L1, GLAST, GLT-1, CD44, and vimentin, are used to label astrocytes. In our study, we only compared the classical cerebral astrocytes labelled by the novel proposed marker NDRG2 and the most commonly used markers: GFAP, S100*β*, and GS in the cerebrum.

In conclusion, our study indicates that NDRG2 and S100*β* are more appropriate markers for astrocytes in the cortex and thalamus, respectively, as well as for observing the overall distribution and changes in the number of astrocytes throughout the cerebrum. Meanwhile, GFAP is superior to NDRG2 and S100*β* when labelling the processes and branches of astrocytes in the corpus callosum, cerebral peduncle, and especially the hippocampus. Our study shows the importance of choosing the most appropriate marker for astrocytes in different mouse cerebral regions, which provides guidance for neuroscience researchers when exploring astrocytic functions.

## Figures and Tables

**Table 1:** Sequence of primers used for real time PCR.

Gene	Forward Primer (5-3′)	Reverse Primer (5-3′)
*Gfap*	GCTGGAGGGCGAAGAAAACCG	CACGGATTTGGTGTCCAGGCTGG
*Ndrg2*	GAGTTAGCTGCCCGCATCC	GTGACCGAGCCATAAGGTGTC
*S100β*	GCTGACCACCATGCCCCTGTAG	CTGGCCATTCCCTCCTCTGTC
*Gapdh*	TGTGTCCGTCGTGGATCTGA	CCTGCTTCACCACCTTCTTGA

**Table 2:** Astrocytic immunoreactivity to NDRG2, GFAP and S100*β* in different cerebral regions.

	GFAP	NDRG2	S100*β*
Cortex (dorsal)	+	+++	+++
Cortex (ventral)	+	++	++
Corpus callosum	++	+	+
Hippocampus	+++	++	++
Thalamus	+	+++	+++
Cerebral peduncle	++	+	+

+++: strongly positive; ++: moderately positive; +: weakly positive.

## Data Availability

The data used to support the findings of this study are included within the article.

## Supplementary Materials

Supplementary Materials Supplementary Figure 1. The colocalization of NDRG2 and GS within astrocytes. Supplementary Figure 2. The expression of NDRG2 protein in cell lines.
