# Supplementary material for: The Appropriate Marker for Astrocytes: Comparing the Distribution and Expression of Three Astrocytic Markers in Different Mouse Cerebral Regions
Source: Biomed Res Int. 2019 Jun 24;2019:9605265. doi: 10.1155/2019/9605265 (PMC6613026; doi:10.1155/2019/9605265)
Supplement: Supplementary Materials — Supplementary Figure 1. The colocalization of NDRG2 and GS within astrocytes. Supplementary Figure 2. The expression of NDRG2 protein in cell lines. [file 9605265.f1.docx]

**Supplementary materials**

***
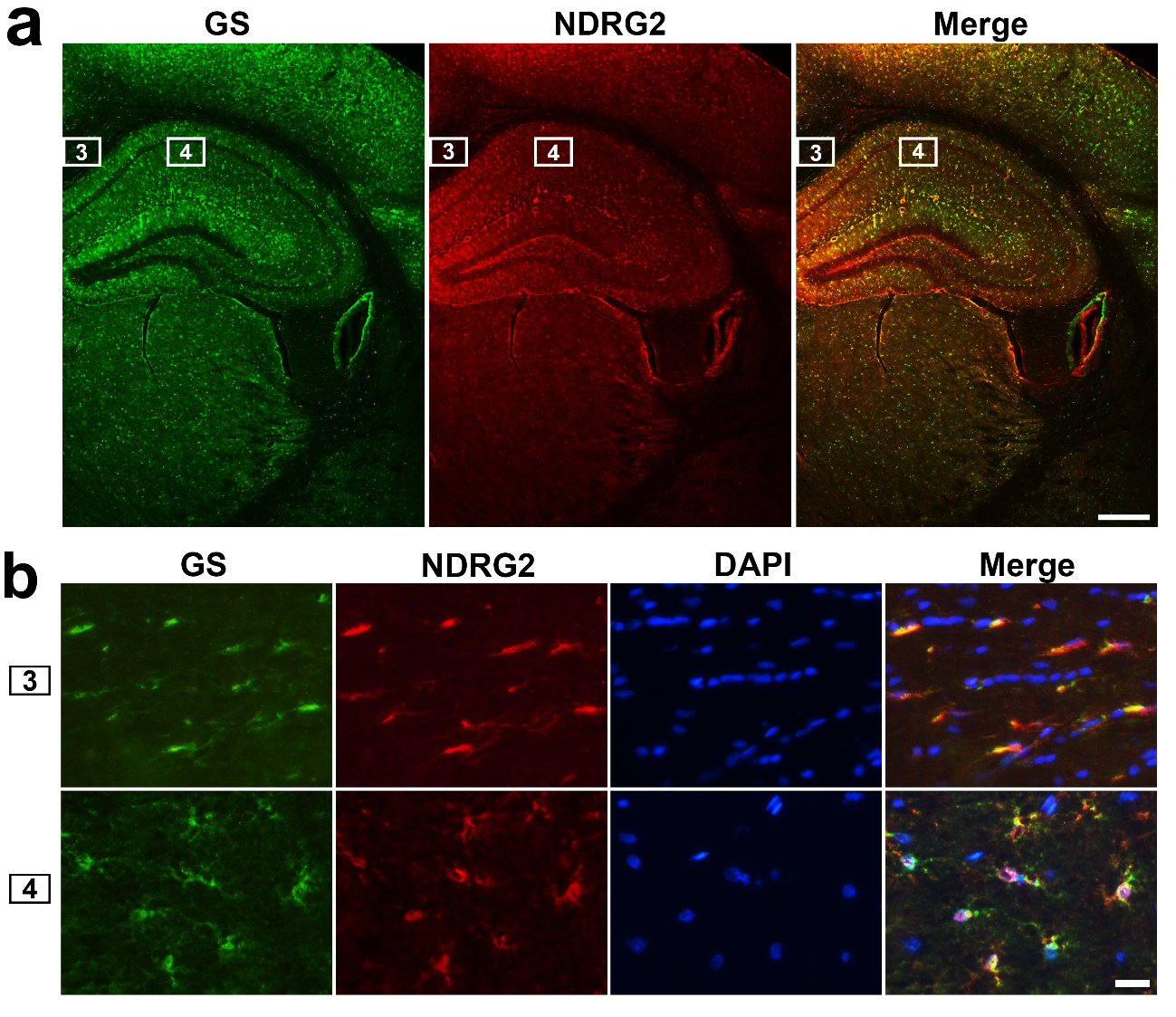
***

***Fig s1 The colocalization of NDRG2 and GS within astrocytes***

a, Representative immunofluorescence images of astrocytes labeled by NDRG2 and GS in the cerebrum of the adult male mice (6 months) at low magnification. Region 3: Corpus callosum; Region 4: Hippocampus. Scale bars=500 µm. b, Representative immunofluorescence images of astrocytes labeled by NDRG2 and GS in the corpus callosum and hippocampus of the adult male mice (6 months). Region 3: Corpus callosum; Region 4: Hippocampus. Scale bars=20 µm.


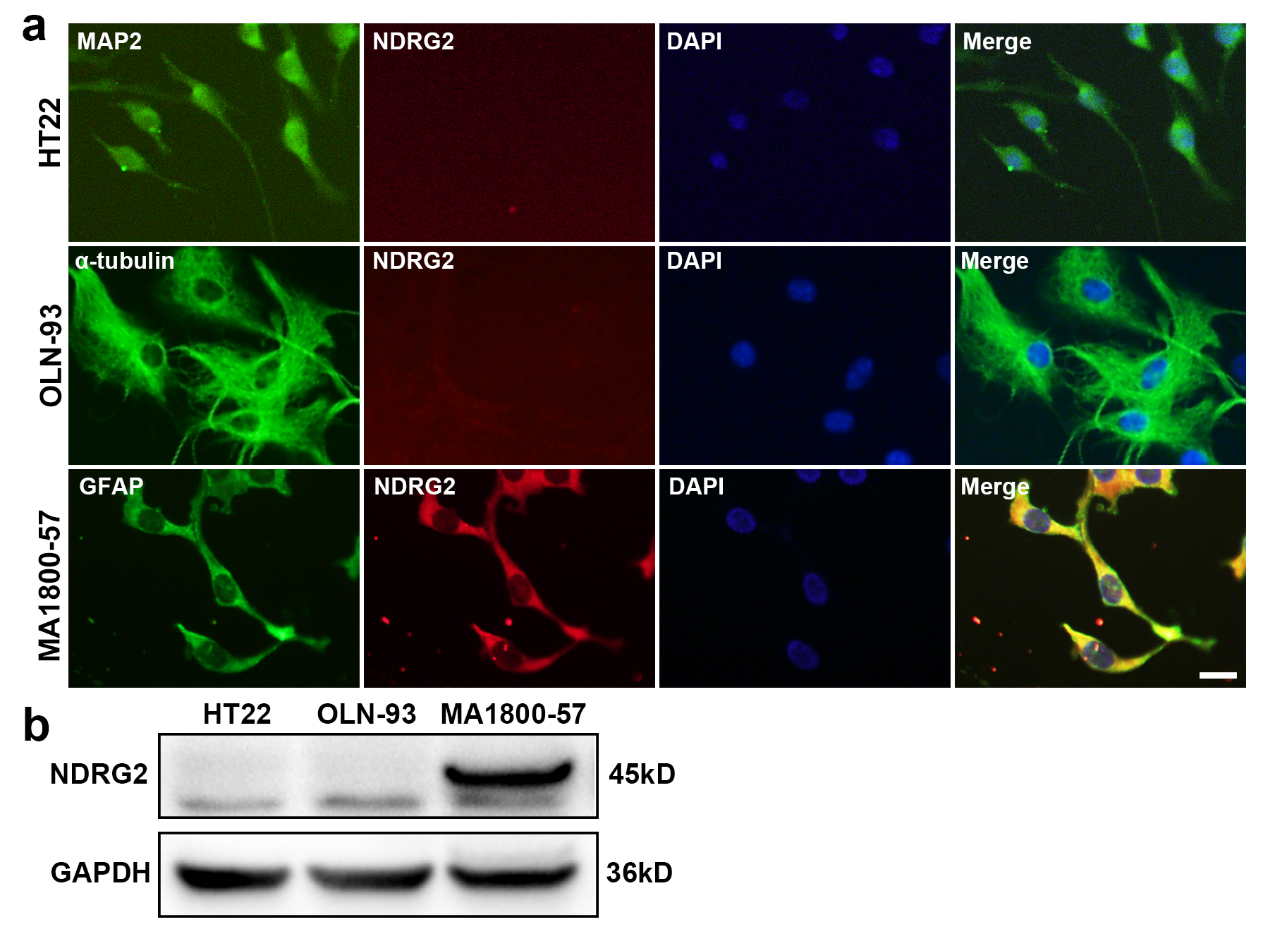


***Fig s2 The expression of NDRG2 protein in cell lines***

a, Representative immunofluorescence images of cell lines. Neuronal cells HT22, oligodendroglial cells OLN-93 and astrocytic cells MA1800-57 were labeled by MAP2, α-tubulin and GFAP, respectively. Scale bars=20 µm. b, The levels of NDRG2 protein in HT22, OLN-93 and MA1800-57 cells were determined by Western blot analysis. Representative blots were shown.
